# The immune regulatory effects of tetrahedral framework nucleic acid on human T cells via the mitogen‐activated protein kinase pathway

**DOI:** 10.1111/cpr.13084

**Published:** 2021-06-25

**Authors:** Xuyang Liu, Zhiyuan Yu, Ya Wu, Sirong Shi, Jie Yao, Xiaorong Feng, Dingke Wen, Ziyan Shi, Zhengyang Zhao, Yanjing Li, Hongyu Zhou, Chao You, Yunfeng Lin, Mu Yang

**Affiliations:** ^1^ Department of Neurosurgery West China Hospital Sichuan University Chengdu China; ^2^ Sichuan Cancer Hospital & Institute Centre for Translational Research in Cancer Sichuan Cancer Center Chengdu China; ^3^ State Key Laboratory of Oral Diseases National Clinical Research Center for Oral Diseases West China Hospital of Stomatology Sichuan University Chengdu China; ^4^ Department of Neurology West China Hospital Sichuan University Chengdu China; ^5^ College of Biomedical Engineering Sichuan University Chengdu China; ^6^ School of Medicine University of Electronic Science and Technology of China Chengdu China

**Keywords:** autoimmunity, immune regulatory, neuromyelitis optica spectrum disorder, T cells, tetrahedral framework nucleic acid

## Abstract

**Objectives:**

Autoimmune diseases are a heterogeneous group of diseases which lose the immunological tolerance to self‐antigens. It is well recognized that irregularly provoked T cells participate in the pathological immune responses. As a novel nanomaterial with promising applications, tetrahedral framework nucleic acid (TFNA) nanostructure was found to have immune regulatory effects on T cells in this study.

**Materials and Methods:**

To verify the successful fabrication of TFNA, the morphology of TFNA was observed by atomic force microscopy (AFM) and dynamic light scattering. The regulatory effect of TFNA was evaluated by flow cytometry after cocultured with CD3+ T cells isolated from healthy donors. Moreover, the associated signaling pathways were investigated. Finally, we verified our results on the T cells from patients with neuromyelitis optica spectrum disorder (NMOSD), which is a typical autoimmune disease induced by T cells.

**Results:**

We revealed the alternative regulatory functions of TFNA in human primary T cells with steady status via the JNK signaling pathway. Moreover, by inhibiting both JNK and ERK phosphorylation, TFNA exhibited significant suppressive effects on IFNγ secretion from provoking T cells without affecting TNF secretion. Similar immune regulatory effects of TFNA were also observed in autoreactive T cells from patients with NMOSD.

**Conclusions:**

Overall, our results revealed a potential application of TFNA in regulating the adaptive immune system, as well as shed a light on the treatment of T cell–mediated autoimmune diseases.

## Graphical abstract

Tetrahedral framework nucleic acid, as a novel nanomaterial, was used in human primary T cells for the first time. TFNA could regulate the immune response of T cells compared with immunosuppressors both in physiological and pathological circumstances via the MAPK signaling pathway. TFNA could regulate the immune response of T cells in peripheral blood of patients with NMOSD ex vivo.

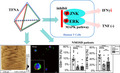

## 1 INTRODUCTION

Autoimmune diseases represent a family of complex diseases, which are characterized by the dysregulation of the host immune system leading to the damage of self‐organs.^1^ As the major components of the adaptive immune system, T cells recognize auto‐antigens and play important roles in promoting antigen‐specific immune responses, thus triggering disease onset. It is well documented that those irregular provoked T cells also associated with cytokine and chemokine cascades, which might cause target organ damages, as well as disease progression and poor prognosis of patients.^2^ Clinically, other than biological therapy, mounts of small‐molecular drugs are currently used in preventing and eliminating T cell–mediated autoimmune responses, including cell proliferation inhibitor cyclosporine A (CsA), IMPDH inhibitor mycophenolate mofetil, and lymphocyte homing inhibitor fingolimod.^3^, ^4^, ^5^ Nevertheless, the above‐mentioned immunosuppressive strategies exhibit a less selective manner and are often found to break the homeostasis of the host immune system.^6^ Therefore, it is urgent to develop an efficient way not only decreases specific immune response induced by autoreactive T cells, but also protects regular host immune responses.

As a novel nanoscale material, tetrahedral framework nucleic acid (TFNA) is composed of four editable isometric single‐strand DNA (ssDNA) molecules, which could self‐assemble and form a stable three‐dimensional DNA nanostructure with excellent endocytotic properties.^7^, ^8^ Additionally, the multi‐biological activities of TFNA have already been revealed.^9^, ^10^, ^11^, ^12^ For instance, TFNA could polarize tissue‐resident macrophages from M1 to M2 phenotypes that promotes the recovery of bisphosphonates‐mediated jaw osteonecrosis in mice.^13^ Similarly, TFNA significantly down‐regulates IL‐1β and IL‐6 secretion by LPS‐induced macrophages in attenuating bacterial inflammation.^14^ Moreover, TFNA was also found to attenuate immune cell recruitment via blocking NF‐κB activation by epithelial cells.^15^ However, in spite of these remarkable functions in modulating innate immune responses at animal models, whether TFNA exhibits similar effects in human adaptive immunity remains unrevealed. Here, we aim to investigate the immune regulatory activities of TFNA in different types of primary human T cells under both physiological and pathological circumstances, and we also corroborate the negative regulatory effect of TFNA on T cells from neuromyelitis optica spectrum disorder (NMOSD) patients.

## 2 EXPERIMENTAL SECTION/METHODS

### 2.1 Synthesis and characterization of tetrahedral framework nucleic acid

The TFNA nanostructure was synthesized based on a previous method.^16^, ^17^ Briefly, four specifically designed S1‐S4 ssDNA molecules (Table 1) were synthesized by Takara Biotechnology. Equal concentrations of four ssDNA molecules were mixed in TM buffer (10 mM Tris‐HCl, pH 8.0; 50 mM MgCl_2_). The buffer was rapidly warmed to 95°C for 10 minutes and then rapidly cooled to 4°C for 20 minutes. The successful synthesis of TFNA was confirmed by 8% polyacrylamide gel and capillary electrophoreses. Dynamic light scattering using a Zetasizer Nano ZS90 (Malvern Instrument Ltd) was applied to examine the average size of TFNA. Atomic force microscopy measurements were performed in the tapping mode using a SPM‐9700 instrument (Shimadzu).

**TABLE 1:** Base sequence of 4 single‐strand DNAs

ssDNA	Base sequence
S1	5′‐ATTTATCACCCGCCATAGTAGACGTATCACC AGGCAGTTGAGACGAACATTCCTAAGTCTGAA
S2	5′‐ACATGCGAGGGTCCAATACCGACGATTACA GCTTGCTACACGATTCAGACTTAGGAATGTTCG
S3	5′ACTACTATGGCGGGTGATAAAACGTGTAGCA AGCTGTAATCGACGGGAAGAGCATGCCCATCC
S4	5′‐ACGGTATTGGACCCTCGCATGACTCAACTGC CTGGTGATACGAGGATGGGCATGCTCTTCCCG

### 2.2 Study population

From April 2019 to May 2020, 10 NMOSD patients and six volunteers were enrolled at the Department of Neurology, West China Hospital, Sichuan University. Patients with NMOSD were included if they met the following criteria: (i) patients were more than 18 years old and (ii) fulfilled the 2015 diagnostic criteria for NMOSD published by the International Panel for NMOSD Diagnosis (IPND).^18^ In contrast, patients with NMOSD were excluded if they had coexisting autoimmune disorders or infections. Clinical data, including sex, age, disease duration, clinical phenotypes, expanded disability status scale (EDSS) score, and serum aquaporin‐4 antibodies (cell‐based assay, CBA), were collected. This study was approved by the Ethics Committee of West China Hospital of Sichuan University, Chengdu, China (No. 2018‐29). Written informed consent forms were obtained from all participants at enrollment.

### 2.3 Isolation of T cells

Peripheral blood mononuclear cells (PBMCs) were isolated from venous blood drawn from healthy volunteers and patients with NMOSD through Ficoll gradient centrifugation (GE Healthcare), according to the supplier's instructions. Subsequently, T cells were isolated using a human CD3+ T‐cell isolation kit (BioLegend). Briefly, non CD3+ T cells were depleted by incubation with a biotin antibody cocktail including anti‐CD14, ‐CD15, ‐CD16, ‐CD19, ‐CD36, ‐CD56, ‐CD123, and ‐CD235, followed by incubation with magnetic streptavidin nanobeads.

### 2.4 Cell culture

T cells were cultured in RPMI medium (Gibco Life Technologies) supplemented with 10% fetal bovine serum (Gibco) at 37°C under conditions of 5% CO_2_ humidified air. T cells were treated with TFNA (250 nM) or CsA (1 µg/mL) for 12 hours. To simulate pathological conditions, T cells were incubated with phorbol myristate acetate (PMA, 100 ng/mL; Biogems) and ionomycin (1 µg/mL; Biogems) for 12 hours.

### 2.5 Cellular uptake of tetrahedral framework nucleic acid

To detect the uptake of TFNA in T cells, TFNA was fluorescently marked by loading cyanine‐5 (Cy5) in one of the four ssDNA molecules. For immunofluorescent staining, T cells were seeded on a polylysine‐treated glass in a 6‐well plate and incubated with TFNA‐Cy5 for 6 hours. After fixing with 4% paraformaldehyde (Biosharp), T cells were washed and stained with phalloidin (Abcam) and DAPI (Abcam). Finally, stained T cells were observed under a confocal microscope (Nikon). For flow cytometric analysis, cells were harvested and measured at different time points using a flow cytometer (FC500 Beckman).

### 2.6 Flow cytometry

Cells were harvested and washed before staining for extracellular markers including CD3, CD4, CD8, CD45RO, CD62L, CD127, and KLRG1 (BioLegend). For intracellular cytokine analysis, cells were stimulated with phorbol myristate acetate, ionomycin, and brefeldin A (Biolegend) for 4 hours. After stimulation, cells were stained with surface antibodies, followed by fixation with the BD fixation/permeabilization solution and staining with intracellular antibodies for IFNγ, TNF, IL‐4, IL‐10, and granzyme B (BioLegend) using 1X perm/wash solution according to the manufacturer's instructions. All samples were washed and analyzed using a FACS Canto II flow cytometer (BD). Data were analyzed using the FlowJo software (Version 10; TreeStar, Ashland).

### 2.7 Western blotting

A whole‐cell lysis kit (KeyGen) was used to harvest the total protein. Consecutively, 10% SDS‐polyacrylamide gel electrophoresis was performed to separate target proteins, which were then transferred to polyvinylidene fluoride membranes. After blocking in 5% bovine serum albumin for 1 hour, target membranes were incubated with anti‐ GAPDH [6C5] (1:1000; Abcam), JNK (1:1000; Cell Signaling Technology), phospho‐JNK [81E11] (1:1000; CST), P38 [D13E1] (1:1000; CST), phospho‐P38 [D3F9] (1:1000; CST), ERK [137F5] (1:1000; CST), and phospho‐ERK [D13.14.4E] (1:1000; CST) primary antibodies overnight at 4°C. After washing with TBST solution (0.1% Tween‐20, 10 mM Tris‐base, and 100 mM NaCl; pH 7), membranes were incubated with an appropriate secondary antibody (1:1000; Abcam) for 1 hour. Finally, the Bio‐Rad enhanced chemiluminescence detection system was used to detect each protein. Quantification was performed by using the ImageJ software.

### 2.8 Statistical analysis

Statistical analyses were performed using GraphPad Prism (Version 6.0). All data were presented as the mean ± SD, with n ≥ 3 replicates. Means for all data were compared using a paired one‐way ANOVA. *P*‐values of <.05 were considered statistically significant: *P < .05, **P < .01, ***P < .001, ****P < .0001.

## 3 RESULTS

### 3.1 Characterization of Synthesized TFNA

**FIGURE 1:**
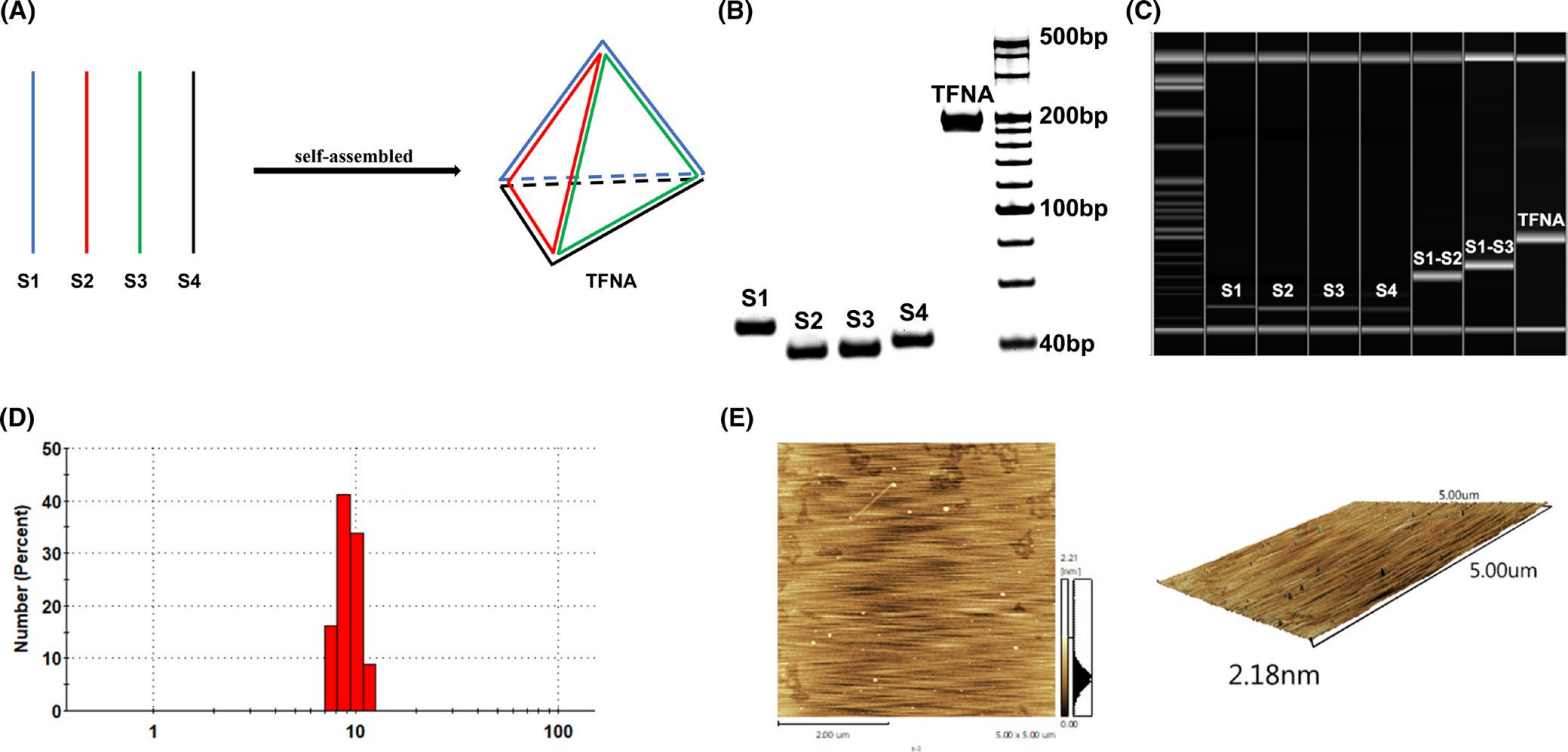
Characterization of TFNA. A, Four isometric single‐strand DNAs formed the stable three‐dimensional structure of TFNA. B, 8% PAGE verify the self‐assembling of TFNA. C, Capillary electrophoresis of four ssDNA molecules, S1‐S2, S1‐S3, and TFNA. D, Size distribution graphs of TFNA. E, Analysis images of TFNA by atomic force microscope

After gently mixing four selected ssDNA molecules in TM buffer, each ssDNA could automatically form a triangle by sharing the sides with other three paired ssDNA molecules and finally form the designated TFNA (Figure 1A). The molecular weights of assembled TFNA were examined by polyacrylamide gel electrophoresis assay and capillary electrophoresis analysis. In consistent with its theoretical structure, the molecule weights of each ssDNA and TFNA are approximately 40 bp and 180 bp, respectively (Figure 1B,C). Moreover, the size distribution and morphology of our generated TFNA were further identified by dynamic light scattering assay and atomic force microscopy (Figure 1D,E). Accordingly, the TFNA was observed to have the particle size of approximately 10 nm with 2.2 nm in height (Figure 1D.E). Altogether, above results confirmed that we successfully obtained the self‐assembled TFNA with designated size and physical morphology.

### 3.2 Identification of the endocytic efficiency

**FIGURE 2:**
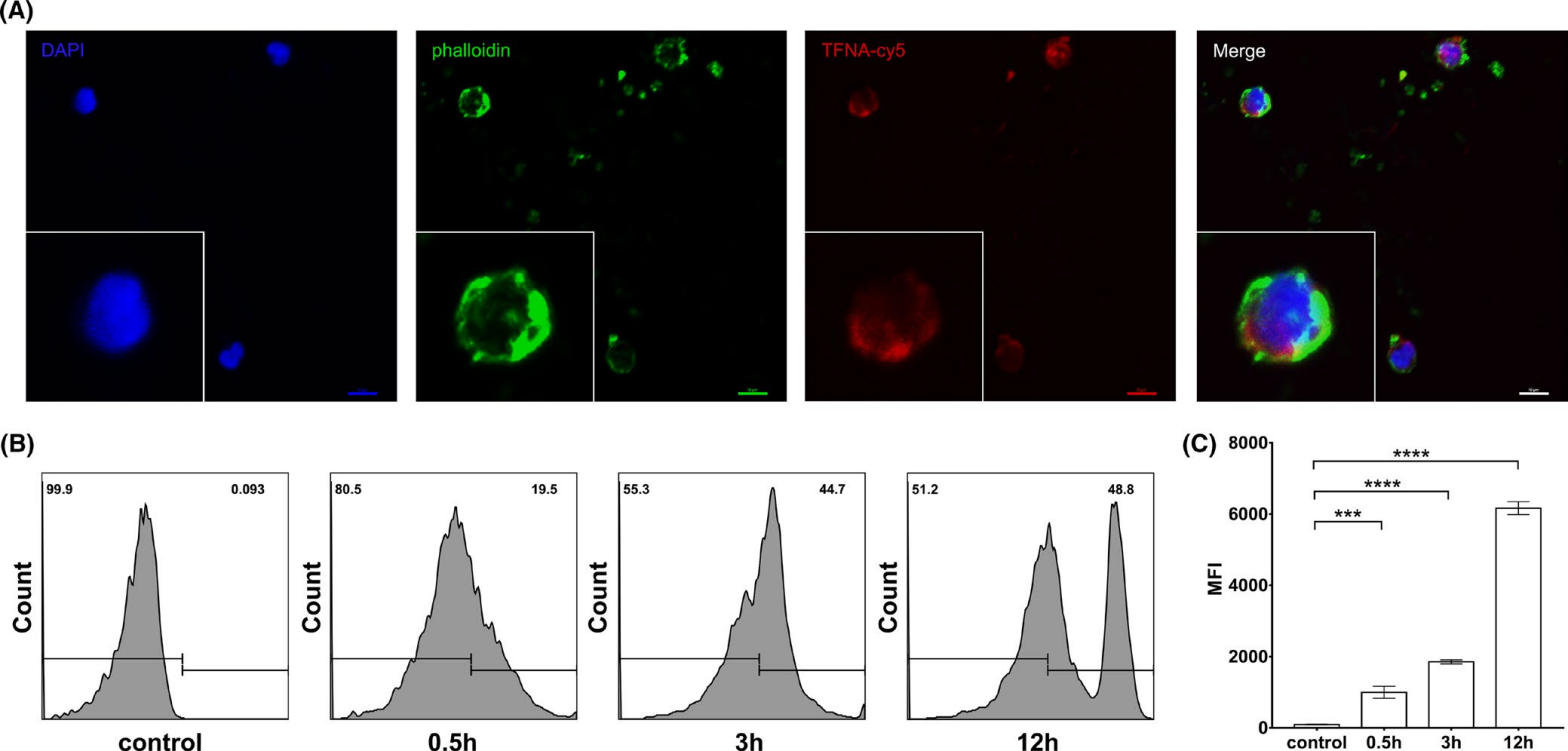
Cellular uptake of TFNA in T cells. A, Endocytosis of TFNA‐cy5 in T cells under an immunofluorescence microscope. (TFNA‐cy5: red; nucleus: blue; cytoskeleton: green). Scale bars: 10 μm. B, Flow cytometric examination of cellular uptake of TFNA‐cy5. C, Semi‐quantitative analysis of cellular uptake in flow cytometry. Data are presented as mean ± SD (n = 3)

Previously studies revealed that TFNA could be rapidly taken up by cells through the caveolin‐dependent pathway and subsequently transport to lysosomes.^19^ To identify the endocytosis of TFNA in T cells, primary human T cells were obtained from peripheral blood of healthy donors and following treated by Cy5‐labeled TFNA (TFNA‐Cy5). After 6 hours co‐cultivation, Cy5 signal could be detected by confocal laser scanning microscopy. As shown in Figure 2A, TFNA‐Cy5 fluorescence signals was observed in the majority of cultured T cells, which were labeled with phalloidin cellular skeleton dye. The efficiency of endocytosis was further revealed by flow cytometry analysis. According to the results, up to 20% of primary human T cells exhibited Cy5 signals at 0.5 hours, whereas the percentile of Cy5 positive cells increased to 45% in total after 3 hours co‐culturing (Figure 2B,C). The median fluorescence intensity (MFI) in T cells increased up to approximately 6000 after cocultured with TFNA for 12 hours. Taken together, TFNA was efficiently taken up by human primary T cells in the absence of a transfection reagent.

### 3.3 IFNγ level was reduced in primary human T cells after TFNA treatment

**FIGURE 3:**
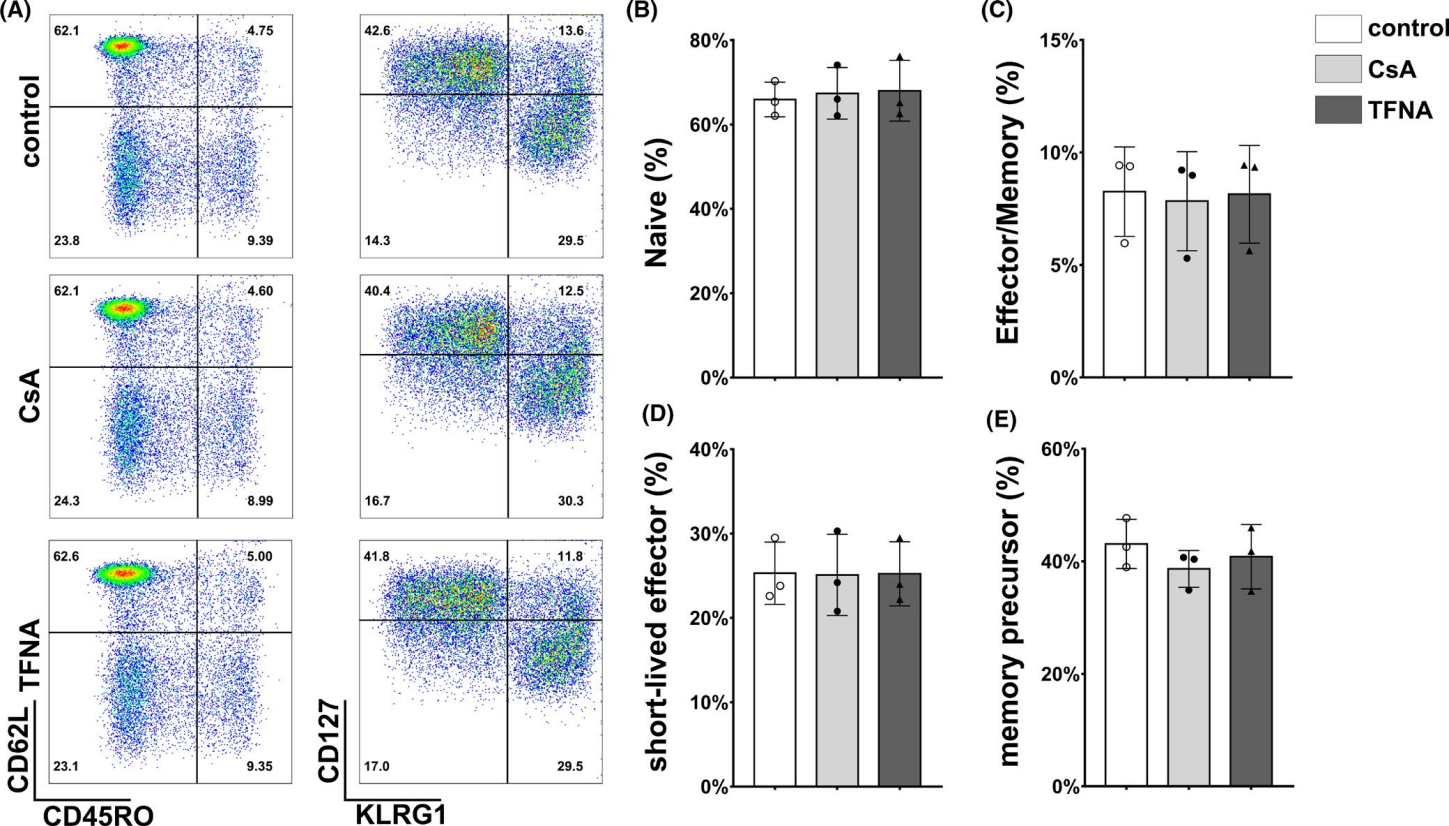
Effect of TFNA and cyclosporine on phenotypes of T cells from healthy donors. A, Representative flow cytometry plots show effect of TFNA and cyclosporine on phenotypes of CD8+ T cells. B, Statistical analyses of TFNA and cyclosporine on naive CD8+ T cells. C, Statistical analyses of TFNA and cyclosporine on Effector/Memory CD8+ T cells. D, Statistical analyses of TFNA and cyclosporine on short‐lived effector CD8+ T cells. E, Statistical analyses of TFNA and cyclosporine on memory precursor CD8+ T cells. Data are presented as mean ± SD (n = 3)

**FIGURE 4:**
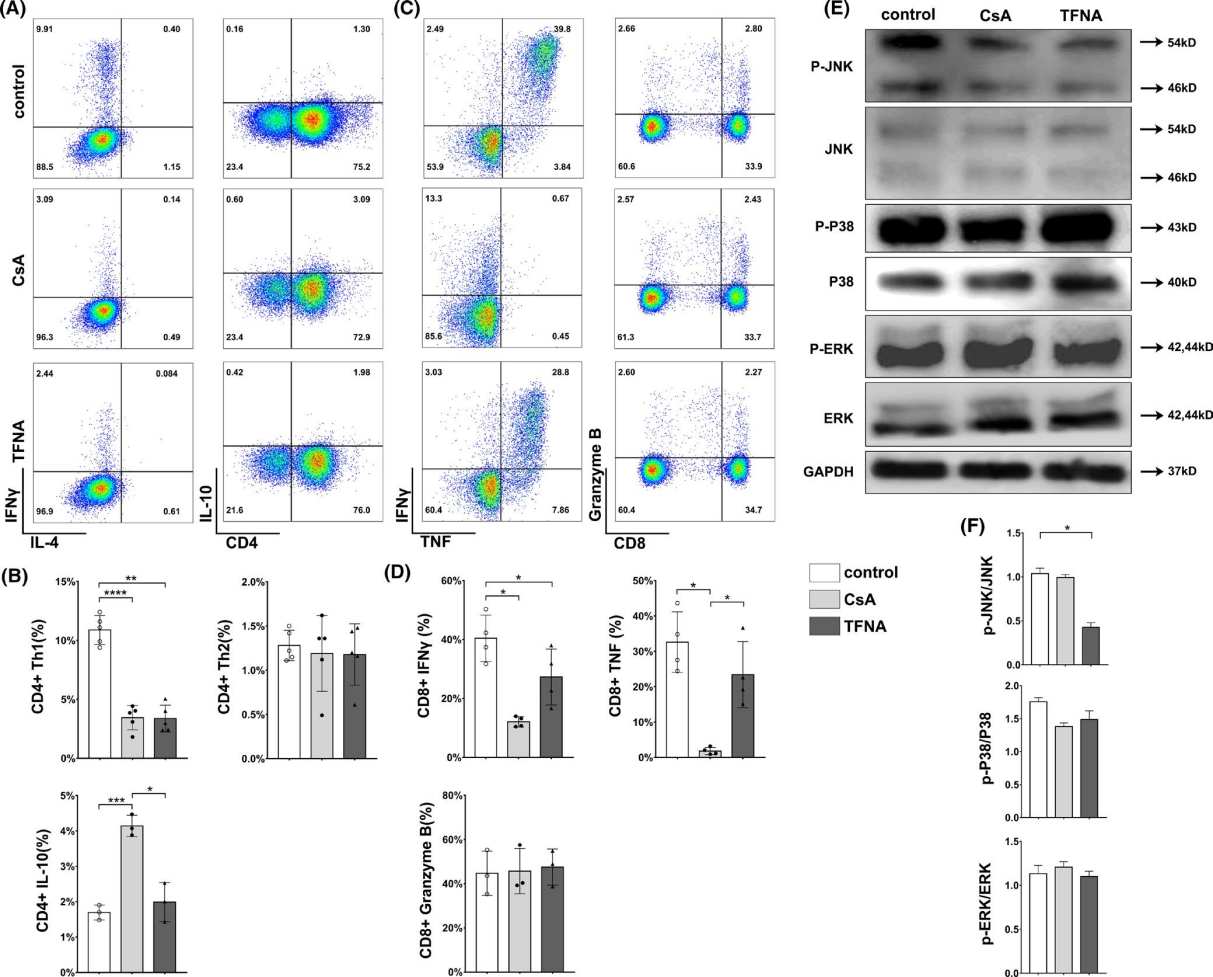
Effect of TFNA and cyclosporine on T cells from healthy donors. A, Representative flow cytometry plots show effect of TFNA and cyclosporine on subtypes of CD4+ T cells. B, Statistical analyses of TFNA and cyclosporine on subtypes of CD4+ T cells. C, Representative flow cytometry plots show effect of TFNA and cyclosporine on intracellular cytokines of CD8+ T cells. D, Statistical analyses of TFNA and cyclosporine on intracellular cytokines of CD8+ T cells. E, Protein expressions of total p38, phospho‐P38, total ERK, phospho‐ERK, total JNK, and phospho‐JNK in T cells were detected by Western blot technique. Lane 1: control group; lane 2: CsA group; lane 3: TFNA group. F, Quantification of the phosphorylation level of JNK, P38, and ERK in different treatment groups. Data are presented as mean ± SD (n ≥ 3)

To investigate whether TFNA could regulate the phenotype and functions of primary human T cells, we conducted an in vitro experiment to evaluate the activities of T cells after TFNA treatment. T cells were sorted using magnetic nanobeads from peripheral blood mononuclear cells (PBMCs) of healthy donors. As a well‐recognized immuno‐suppressor, CsA was used to treat T cells as control, and several markers were employed to distinguish subtypes of CD4+ and CD8+ T cell, including IFNγ for Th1, IL‐4 for Th2 cells and IL‐10 for Treg cells in CD4+ T cell population, as well as naïve T (T_N_) cells (CD62L+/CD45RO‐), effector memory T(T_E/M_) cells (CD62L‐/CD45RO+), short‐lived effector T (T_SLE_) cells (KLRG1+/CD127‐) and memory precursor effector T (T_MP_) cells (KLRG1‐/ CD127+) in CD8+ T cell population.^20^ After 12 hours cultivation, the percentile of T_N_, T_E/M_, T_SLE_ and T_MP_ CD8+ T cells were detected as well (Figure 3A), no significantly change of above‐mentioned subsets was observed among control, CsA or TFNA treatment groups (Figure 3B‐E). On the other hand, as shown in Figure 4A, compared with the control group, TFNA exhibited immune inhibitory effect in decreasing the percentile of the Th1 cell subgroup, but not the Th2 cell subgroup. However, the elevation of the Treg cell subgroup was only monitored in the CsA‐treated group (Figure 4B). Overall, TFNA down‐regulated IFNγ secretion from T cells without changing their phenotypes. Nevertheless, despite the surface marker expression, CsA showed a robust effect in eliminating both IFNγ and TNF secretion from CD8+ T cells (Figure 4C), but TFNA only contributed to decrease the IFNγ level (Figure 4D). Additionally, granzyme B expression in CD8+ T cells was affected by neither TFNA nor CsA (Figure 4D). Taken together, TFNA displayed remarkable regulatory roles in restraining IFNγ secretion in both CD4+ and CD8+ T cells at steady status.

### 3.4 Tetrahedral framework nucleic acid downregulates IFNγ secretion by inhibiting JNK phosphorylation

We further investigated the phosphorylation levels of ‐Jun N‐terminal kinase (JNK), p38 and extracellular signal‐regulated kinase (ERK; Figure 4E,F). It is well‐recognized that JNK, p38, and ERK belong to the mitogen‐activated protein kinase (MAPK) family, which orchestrates cytokine secretion, T cell activation and differentiation during adaptive immune responses. Interestingly, except the phosphorylated JNK level was inhibited by TFNA, the phosphorylation levels of both ERK and p38 were not affected (Figure 4E,F). Therefore, our presence results illustrated that the immune‐inhibitory roles of TFNA were partially dependent on blocking JNK phosphorylation in primary human T cells.

### 3.5 Tetrahedral framework nucleic acid inhibited primary human T cell activation

**FIGURE 5:**
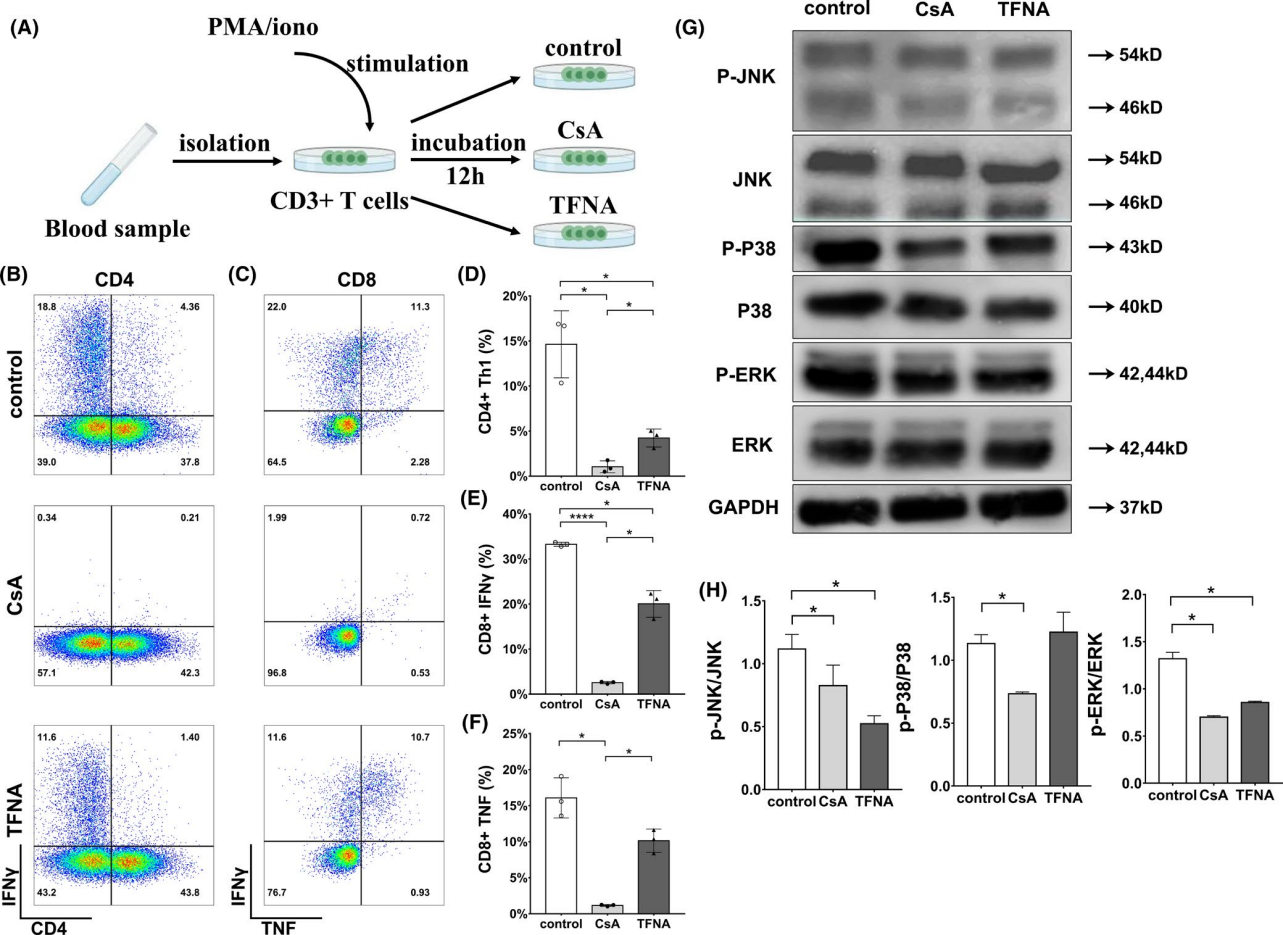
Effect of TFNA and cyclosporine on T cells under pathological circumstances. A, Schematic description of procedures to mimic the pathological circumstances by stimulating human primary T cells. B, Representative flow cytometry plots show effect of TFNA and cyclosporine on subtypes of CD4+ T cells under pathological circumstances. C, Representative flow cytometry plots show effect of TFNA and cyclosporine on intracellular cytokines of CD8+ T cells under pathological circumstances. D, Statistical analysis of Th1 group of CD4+ T cells under pathological circumstances. E, Statistical analysis of IFNγ+ group of CD8+ T cells under pathological circumstances. F, Statistical analysis of TNF+group of CD8+ T cells under pathological circumstances. G, Protein expressions of total p38, phospho‐P38, total ERK, phospho‐ERK, total JNK and phospho‐JNK in T cells under pathological circumstances were detected by western blot. Lane 1: control group; lane 2: CsA group; lane 3: TFNA group. H, Quantification of the phosphorylation level of JNK, P38, and ERK in different treatment groups. Data are presented as mean ± SD (n = 3)

T cells stimuli PMA and ionomycin were then employed to prime primary human T cells for 12 hours in mimicking the pathological circumstances (Figure 5A). Consistent with our previous results, TFNA successfully suppressed the pro‐inflammatory cascades of activated T cells (Figure 5B‐F). In detail, IFNγ was sharply reduced in CD4+ T cells after TFNA treatment (Figure 5B, D). Simultaneously, the IFNγ level in CD8+ T cells was found to decrease after TFNA 12 hours treatment (Figure 5C, E,F). It is noted that a relatively successive immune‐inhibitory effect of TFNA was observed on provoked T cells in comparing with T cells without stimulation (Figures 4B, D and 5B‐F). In line with our observations above (Figure 4E,F), the phosphorylation of JNK in provoked T cells was blocked by TFNA (Figure 5G,H). Moreover, a significant down‐regulation of the ERK phosphorylation level was detected in the TFNA group as well (Figure 5G,H). Notably, phosphorylation levels of JNK, P‐38 and ERK were significantly downregulated in provoked T cells after treated with CsA, which showed no effect on MAPK signaling in T cells at steady status (Figure 4E,F and 5G,H). Altogether, our observations demonstrated an ERK/JNK–dependent constitutively inhibitory manner of TFNA in T‐cell activation under pathological circumstances and suggesting that TFNA could be considered as an immune regulator in preventing T cell–mediated inflammation.

### 3.6 Tetrahedral framework nucleic acid exhibits regulatory effects in eliminating activated T cells from patients with Neuromyelitis optica spectrum disorder

**FIGURE 6:**
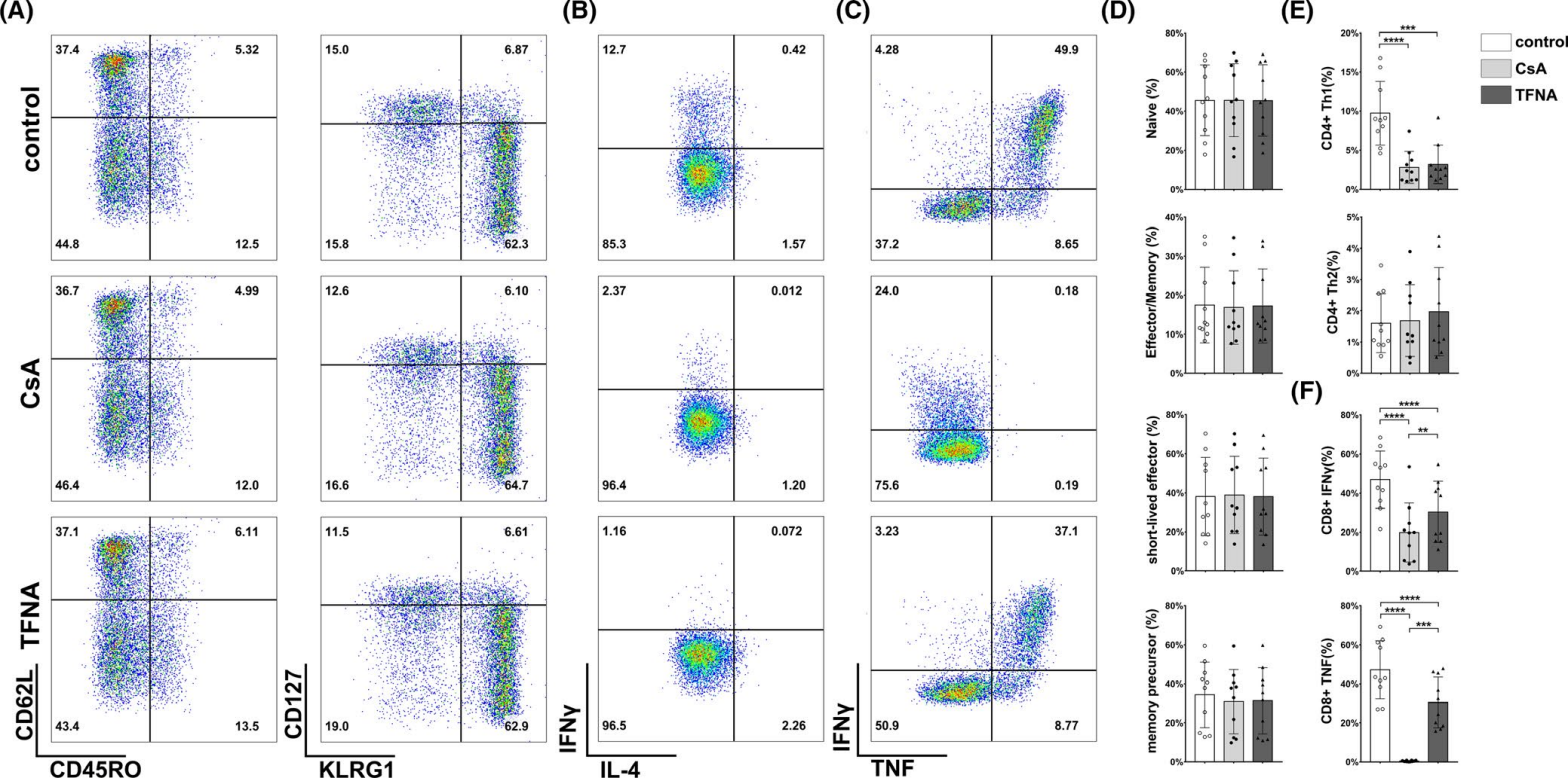
Effect of TFNA and cyclosporine on T cells in NMOSD patients. A, Representative flow cytometry plots show effect of TFNA and cyclosporine on phenotypes of CD8+ T cells in NMOSD patients. B, Representative flow cytometry plots show effect of TFNA and cyclosporine on subtypes of CD4+ T cells in NMOSD patients. C, Representative flow cytometry plots show effect of TFNA and cyclosporine on intracellular cytokines of CD8+ T cells in NMOSD patients. D, Statistical analyses of different phenotypes of CD8+ T cells in NMOSD patients. E, Statistical analyses of different subsets of CD4+ in NMOSD patients. F, Statistical analyses of different subsets of CD8+ T cells in NMOSD patients. Data are presented as mean ± SD (n = 10)

Neuromyelitis optica spectrum disorder represents a relapsing autoimmune disease that preferentially affects the central nervous system including the brain, optic nerve and spinal cord where patients suffer from severe visual impairment and motor disability.^21^ Recent studies indicated that autoreactive T cells played important roles in NMOSD relapsing and progression.^22^ Here, in order to further dissect the regulatory roles of TFNA, T cells were sorted from peripheral blood of admitted NMOSD patients with the acute phase from April 2019 to January 2020 at the Department of Neurology, West China Hospital (Table 2). In consistent with our previously studies, after 12 hours ex vivo cultivation, TFNA illustrated a robust effect in blocking IFNγ secretion from Th1 cells, whereas Th2 cell population remained virtually unchanged among three groups (Figure 6B, E). Meanwhile, although TFNA failed to affect the phenotypes of CD8+ T cells from patients (Figure 6A, D), the IFNγ and TNF secretion levels of CD8+ T cells were dramatically reduced after 12 hours co‐cultivation (Figure 6C, F). It is worth mentioning that a less intensive impact of TFNA in TNF secretion from CD8+ T cells was observed by comparing with the CsA group, which decreased approximately 95% of TNF secretion (Figure 6F). Indeed, this finding emerged a distinct manner of TFNA from currently immunosuppressive‐therapeutic strategies, including both CsA and steroids, which decrease all cytokines and/or chemokines from T cells. In summary, TFNA could serve as a more manageable immune modulator that not only controls disease progression, but also protects host immune responses.

**TABLE 2:** Demographic and clinical characteristics of patients with NMOSD

No.	Gender	Age	Stage	Disease duration (mo)	AQP4 antibody	EDSS score
1	Female	23	Acute stage	6	Positive	3
2	Female	53	Acute stage	102	Positive	8
3	Male	34	Acute stage	1	Negative	3
4	Female	63	Acute stage	35	Positive	4
5	Female	60	Acute stage	7	Positive	6
6	Female	48	Acute stage	2	Positive	2.5
7	Male	65	Acute stage	13	Positive	7
8	Female	36	Acute stage	5	Positive	6
9	Female	55	Acute stage	6	Positive	7.5
10	Female	45	Acute stage	1	Positive	3

Abbreviations: AQP4, Aquaporin‐4; EDSS, Expanded Disability Status Scale; NMOSD, Neuromyelitis optica spectrum disorder.

## 4 DISCUSSION

As documented players in multi‐autoimmune diseases, T cells are considered in participating the progression of both systemic and local inflammation.^23^ However, except circulating autoreactive T cells, CD4+ and CD8+ T cells are also necessary for host immune responses to prevent pathogen infections.^24^ Therefore, to focus a debate regarding the elimination of autoimmune responses and protection of the host immune system, the immune regulatory roles of TFNA were investigated in human primary circulating T cells. A significant reduction in IFNγ secretion was observed in TFNA treated CD4+ and CD8+ T cells with steady status, respectively (Figure 4). Meanwhile, a similar immune suppressive effect of TFNA was observed in primed T cells as well (Figure 5). It is also noteworthy that TFNA had no effect on T cell differentiation at neither primed nor resting T cells (Figure 3). Additionally, our ex vivo studies revealed a significantly blockade of IFNγ and TNF secretion in T cells from NMOSD patients after TFNA treatment (Figure 6). By using T‐cell activation inhibitor CsA as a positive control, we further dissected that TFNA down‐regulated JNK/ERK phosphorylation to block MAPK signaling pathway–dependent cytokine secretion (Figures 4, 5). Altogether, our current findings suggested that TFNA could be used as a novel immune regulator to prevent excessive pro‐inflammatory cytokine secretion from provoked T cells, which is supposed to be the major signs of autoimmune responses.

Due to the unrevealing mechanisms of how T cells participate in autoimmune disease progression, current frontline therapeutic strategies for such diseases are based on systemic immunosuppression, which not only ameliorates disease progression in patients, but also leads to numerous side effects associated.^25^ For instance, corticosteroid hormone might induce severe infection via hamper immune‐cell maturation and differentiation.^26^ Other than renal function damage, immunotherapy cohorts have already demonstrated that CsA increases the risks of leukemia and dermatoma by restraining certain T‐cell populations.^27^ Besides, FK506 (Tacrolimus) conditionally damages the host immune response, as it displays an excellent effect in blocking the IL‐2 signaling pathway.^28^ Unlike those traditional immune suppressors mentioned above, TFNA illustrates a minor influence in metabolism, circulatory system, enzyme catalytic activities and other risks of severe diseases.^29^, ^30^, ^31^ More importantly, by only eliminating cytokine secretion of T cells, TFNA failed to regulate T cell subsets, such as naïve and memory precursor T cells, which diminished the potential effect for host immunity. On account of this selective manner in modulating T‐cell immunity, we speculated that the alternative strengths of TFNA in blocking the MAPK signaling pathway at T cells with different statuses may contribute to this phenomenon. Nevertheless, more detailed information should be discovered in the following studies.

Neuromyelitis optica spectrum disorder is a typical autoimmune disease that affects the central nerve system and T cells play pivotal roles in disease relapsing.^32^ In particular, the Th1/Th2 ratio in the relapsing phase of NMOSD patients was higher than that in NMOSD patients in the remitting phase.^33^ Our latest results demonstrated that circulating CD8+ T cells produce IL‐2 and IFNγ, which are important cytokines for NMOSD relapsing and play crucial roles in disease progression as well.^20^ Unfortunately, although current immunosuppressive therapies concomitant with glucocorticoids are considered as the frontline treatments for controlling the progression of NMOSD, there were still 30%‐53% of patients with advanced disease or relapsing.^34^ Simultaneously, many NMOSD patients have to suffer severe infection due to extremely low host immunity during the treatment.^35^ Hence, it is urgent to develop novel strategies in targeting activated T cells of patients with NMOSD. Here, by involving NMOSD patients, ex vivo studies were designed to further confirm the selective regulatory manners of TFNA in inhibiting both IFNγ and TNF secretion by T cells. Indeed, because of the small sample size, more NMOSD patients are needed to be involved in our further cohort. In addition, accumulating evidence has already mentioned that effect/memory and short‐lived effector T cells lead to autoimmune responses in situ.^36^ Thus, we plan to look insight into the regulatory effects of TFNA in autoantigen‐specific T cells with different subsets. Overall, we illustrated the immune regulatory function of TFNA by downregulating the expression of IFNγ in human T cells without affecting the phenotype and TNF secretion levels. In addition, it showed a more selective manner in modulating the immunity than the immunosuppressors, which made us believe that TFNA could be used as an immunoregulator in the treatment of T cell–mediated autoimmune diseases, including NMOSD and other diseases.

## 5 CONCLUSION

Self‐assembled TFNA was found to be capable of entering and accumulating in the cytoplasm of primary human T cells. On the basis of this finding, we revealed that TFNA regulates T cell‐mediated adaptive immunity in physiological conditions with MAPK signaling pathway–dependent manner. We further revealed that TFNA has a consistently regulatory effect on provoked T cells under pathological circumstances. Moreover, the ex vivo studies demonstrated that TFNA possessed significantly immune‐regulatory roles in inhibiting pro‐inflammatory cytokine secretion, but not changing T‐cell phenotypes in NMOSD patients. To our knowledge, it was the first time to prove the regulatory effects of TFNA in human primary T cells. Our results illustrated that TFNA could be used as a potential immune regulator in the treatment of NMOSD or other T cell–mediated diseases.

## CONFLICT OF INTEREST

The authors declare no conflict of interest.

## AUTHOR CONTRIBUTIONS

X. Liu, S. Shi, Z. Shi and Y. Li performed the experiments. Y. Wu, J. Yao, X. Feng and Z. Zhao analyzed the data. X. Liu, Z. Yu and D. Wen wrote the manuscript. H. Zhou, C. You, Y. Lin and M. Yang designed the project and revised the manuscript. All authors have read and agreed to the published version of the manuscript.

## Data Availability

The data that support the findings of this study are available from the corresponding author upon reasonable request.
